# Impacts of Single and Sequential Enzymatic Extraction on the Functional Properties of Khao Dawk Mali 105 Rice Bran Proteins at Two Maturity Stages

**DOI:** 10.3390/foods15030419

**Published:** 2026-01-23

**Authors:** Tarathep Siripan, Apichaya Bunyatratchata, Wanida Chuenta, Jiranan Ratseewo, Hua Li, Sirithon Siriamornpun

**Affiliations:** 1 Department of Food Technology and Nutrition, Faculty of Technology, Mahasarakham University, Kantarawichai, Maha Sarakham 44150, Thailand; 67010853501@msu.ac.th (T.S.); apichaya.b@msu.ac.th (A.B.); wanida.ch@msu.ac.th (W.C.); 2 Research Unit of Thai Food Innovation (TFI), Mahasarakham University, Kantarawichai, Maha Sarakham 44150, Thailand; 3 Division of Food Innovation and Technology, Faculty of Liberal Arts and Science, Sisaket Rajabhat University, Sisaket 33000, Thailand; jiranan.r@gmail.com; 4 Department of Cuisine and Nutrition, Yangzhou University, Yangzhou 225127, China; lihua216@yzu.edu.cn; 5 Key Laboratory of Chinese Cuisine Intangible Cultural Heritage Technology Inheritance, Ministry of Culture and Tourism, Yangzhou 225127, China

**Keywords:** plant protein, protease, α-amylase, extractability, antioxidant activity, structural characterization

## Abstract

Proteins from the bran of Khao Dawk Mali 105 rice at two maturity stages, green (GB) and fully ripe (RB), were extracted using single and sequential enzyme-assisted processes. Non-enzymatic extraction (control), α-amylase (AA), protease (PT), and two sequential treatments (AA-PT and PT-AA) were applied to defatted bran to evaluate their effects on protein yield, structural attributes, and functional properties. Protease-based extractions, particularly PT, produced the highest protein contents (28% in GB and 23% in RB) and significantly improved solubility, water- and oil-holding capacities, and foaming performance. GB extracts consistently outperformed RB across all functional and antioxidant measurements, indicating greater extractability and bioactive potential in green rice bran. Enzymatic hydrolysis also enhanced phenolic and flavonoid release, leading to markedly higher DPPH and FRAP activities. SDS-PAGE profiles demonstrated reduced band complexity and lower-molecular-weight protein in enzymatically treated samples, while FTIR spectra confirmed secondary structural modifications associated with hydrolysis. Overall, protease and sequential assisted extractions provide an efficient and sustainable approach to improving rice bran protein recovery and functionality. These findings highlight green rice bran as a promising source of high-value plant proteins for food and nutraceutical applications.

## 1. Introduction

Rice (*Oryza sativa* L.) is one of the world’s most important staple crops, providing essential energy and nutrients for more than half of the global population [1]. Beyond its role as a primary food source, rice processing generates valuable by-products with significant nutritional potential. Among these, rice bran, the outer layer removed during the milling of rice, accounts for approximately 10% of the weight of rough rice yet contains a disproportionately high concentration of nutrients [2]. Rice bran is notably abundant in proteins, lipids, dietary fiber, vitamins, and antioxidants, rendering it a promising ingredient for food and nutraceutical applications. Despite its nutritional richness, it remains underutilized, often relegated to low-value uses or discarded [3,4]. The bioactive constituents, rice bran protein, have gained increasing attention due to their balanced amino acid composition, hypoallergenic nature, and favorable techno-functional properties such as emulsification, foaming, and solubility [5]. Khao Dawk Mali 105 (KDML 105), commonly referred to as Thai Hom Mali rice, ranks among Thailand’s most commercially important rice varieties. Its characteristic aroma, largely due to 2-acetyl-1-pyrroline, contributes to its premium market value. Given its wide cultivation, substantial amounts of bran are generated but remain largely underutilized. This surplus presents an opportunity to develop rice bran protein as a sustainable, plant-based alternative to conventional protein sources [6]. However, its commercial utilization remains limited. A major constraint lies in the entrapment of proteins within a complex bran matrix of fiber, lipids, and antinutritional factors such as phytates and trypsin inhibitors, which impede extraction efficiency and digestibility [7]. Addressing these challenges aligns with the global shift toward circular bioeconomy principles, waste minimization, and sustainable protein sourcing [8]. Recovering rice proteins efficiently remains challenging due to the strong binding between proteins and starch in the endosperm. Although alkaline extraction typically provides relatively high protein yields, it often results in residual reagents such as NaOH, NaCl, and HCl, introduced during neutralization and subsequent isoelectric precipitation at pH 4.3 [9]. Recent studies have investigated alternative and environmentally friendly extraction methods, particularly enzymatic hydrolysis, which show promise in enhancing protein yield while preserving structural integrity, bioactivity, and functionality [10]. Enzyme-assisted extraction is increasingly recognized as an environmentally sustainable approach for recovering biomolecules [11]. In this process, hydrolytic enzymes degrade starch components and cell wall materials, thereby enhancing protein release under low-severity processing conditions that help preserve the molecular integrity of the proteins. A range of proteases and carbohydrate-degrading enzymes, including α-amylase [12], have been employed to improve extraction efficiency while maintaining the native characteristics of the proteins [13].

However, the conditions of enzymatic extraction, particularly the substrate-to-enzyme ratio and treatment sequence, play a critical role in determining extraction efficiency and protein quality. Despite this, limited information is available on how different enzyme application orders (AA → PT vs. PT → AA) affect the structure–function relationship of rice bran proteins at varying maturity stages. The present study, therefore, aims to evaluate and optimize green enzymatic extraction strategies for efficient protein recovery, with emphasis on maximizing yield, preserving structural integrity, and enhancing techno-functional properties. In addition, it seeks to clarify how key extraction parameters influence protein functionality, thereby supporting the use of rice bran protein as a sustainable, high-performance plant-based ingredient for food and nutraceutical applications.

## 2. Materials and Methods

### 2.1. Materials and Chemicals

KDML 105 rice bran samples were harvested at two developmental stages: green rice bran (GB) collected at approximately 22–28 days after pollination, and fully ripe rice bran (RB) obtained around 29–35 days after pollination. All bran samples were sourced from Suwannaphum, Roi Et Province, Thailand, and immediately stored at 4 °C until further use. The enzymatic materials used in this study included food-grade α-amylase (3000 IU/mL; EC 3.2.1.1; optimal activity at pH 5.5–8.0) and protease (80,000 IU/mL; EC 3.4.21.62; active at pH 3.5–6.5), both provided by KANEZYME, Bangkok, Thailand. Sodium hydroxide (food-grade) was obtained from Krungthepchemi (Bangkok, Thailand), and all analytical-grade reagents, HPLC-grade solvents, and amino acid standards (purity ≥ 90%) were supplied by Sigma-Aldrich (St. Louis, MO, USA).

### 2.2. Extraction of Rice Bran Protein Using Enzymatic Methods

#### Rice Bran Protein Extraction

**Figure 1:**
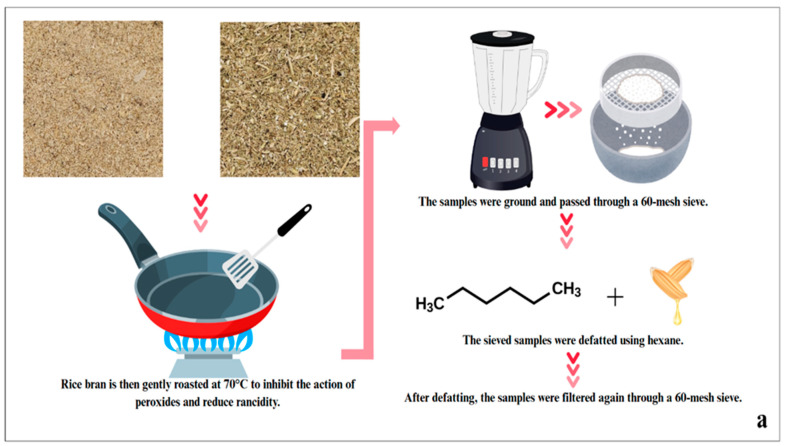

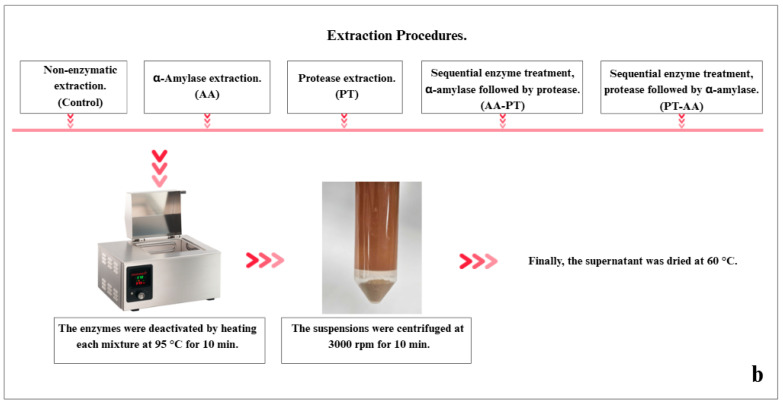
Protein extraction procedure from KDML 105 rice bran samples at two growth stages. (**a**) Sample preparation steps of rice bran prior to extraction; (**b**) Non-enzymatic and enzymatic extraction procedures for rice bran protein.

Rice bran was defatted following the procedure described by Wattanasiritham et al. (2016) [14], with minor adjustments. To reduce peroxide activity and limit rancidity, the bran was lightly roasted at 70 °C [15], passed through a 60-mesh sieve, and extracted with hexane at a 1:3 (*w*/*v*) ratio. The solvent was removed by overnight evaporation under a fume hood, followed by drying to ensure complete hexane removal. The dried material was then sieved again through a 60-mesh screen. The overall sample preparation workflow is shown in Figure 1a.

Five extraction approaches were applied to the rice bran protein: a control without enzymes, α-amylase (AA), protease (PT), and two sequential treatments combining both enzymes (AA followed by PT, and PT followed by AA), following the methods of Siripan et al. [16]. Each treatment involved mixing 50 g of defatted bran with 500 mL of distilled water, followed by pH adjustment to 6.5 with 1 N NaOH or HCl as necessary. Suspensions were incubated at 50 °C with shaking (150 rpm). The control was extracted for 1 h without enzymes. For single-enzyme treatments, α-amylase (0.5% *v*/*v* or 0.52% *w*/*w*; 3000 IU/mL) or protease (0.4% *v*/*v* or 0.42% *w*/*w*; 80,000 IU/mL) was added and allowed to react for 1 h. Sequential treatments consisted of two 30-min incubations in the order AA-PT or PT-AA. All enzymatic reactions were stopped by heating the mixtures at 95 °C for 10 min, followed by cooling to 25 °C. The samples were centrifuged at 3000 rpm for 10 min, and the resulting supernatants were dried at 60 °C to constant weight. A schematic of the extraction procedure is presented in Figure 1b.

### 2.3. Evaluation of Physicochemical Characteristics

#### 2.3.1. Color Measurement

The CIE L*, a*, and b* color parameters were measured using a Chroma Meter (CR-410, Konica Minolta Sensing Inc., Osaka, Japan) following the procedure described by Siripan et al. [16].

#### 2.3.2. Water Activity (a_w_)

An AquaLab meter (Decagon Devices, Pullman, WA, USA) was used to determine water activity (a_w_) following the manufacturer’s guidelines.

#### 2.3.3. Determination of Water Holding Capacity

Water holding capacity (WHC) was assessed using the procedure described by Miedzianka et al. (2023) [17], with slight modifications. Briefly, 0.5 g of sample was mixed with 20 mL of distilled water and subjected to centrifugation at 4000 rpm for 10 min at 10 °C. The weight of the resulting residue was recorded, and WHC was calculated as the proportion of the initial sample weight (W_initial_) to the final weight after water absorption (W_final_), as described in Equation (1).

WHC (g/g) = W_initial_ (g) − W_final_ (g)/W_initial_ (g) (1)

#### 2.3.4. Determination of Oil Holding Capacity

Oil Holding Capacity (OHC) was determined according to the procedure of Miedzianka et al. (2023) [17], replacing water with rice bran oil. OHC was calculated using the same formula as WHC as shown in Equation (2).

OHC (g/g) = W_initial_ (g) − W_final_ (g)/W_initial_ (g) (2)

#### 2.3.5. Measurement of Protein Solubility Content (%)

Protein solubility was determined according to the procedure described by Miedzianka et al. (2023) [17], with minor adjustments. Briefly, 0.25 g of sample was suspended in 25 mL of distilled water (pH 7.0) and agitated for 1 h at a temperature of 25 °C. The mixture was then centrifuged for 20 min at a speed of 4000 rpm, and the resulting supernatant was collected and dried at 105 °C until reaching a stable weight. Protein solubility was calculated using Equation (3).

Solubility content (%) = (W_final_ (g)/W_initial_ (g)) × 100 (3)

#### 2.3.6. Assessment of Foaming Capacity and Stability

Foaming properties were determined using the procedures described by Boonarsa et al. (2024) and Mishyna et al. (2019) [18,19], with minor adjustments. A 1% (*w*/*v*) protein solution (pH 7) was homogenized for 2 min. Foam height was recorded immediately (H_0_) and after 20 min (H_t_). Foaming capacity (FC_0_ at 0 min and FC at 20 min). Foaming capacity and stability were calculated using the following Equations (4) and (5):

Foaming capacity (%) = (H_t_ × H_0_/H_0_) × 100 (4)

Foaming stability (%) = (FC/FC_0_) × 100 (5)

#### 2.3.7. Determination of Moisture

Moisture content was determined using the AOAC Official Method [20] and expressed on a dry-weight basis.

### 2.4. Analysis of Bioactive Compounds and Antioxidant Activities

#### 2.4.1. Extraction of Bioactive Compounds

Bioactive compounds were extracted according to Thammapat et al. (2015) [21]. 1 g of protein extract was mixed with 20 mL of 80% methanol, shaken at 37 °C (150 rpm) for 20 h, and centrifuged at 4000 rpm for 10 min. The supernatant was used for analysis.

#### 2.4.2. Determination of Bioactive Compounds

Total phenolic content (TPC) was measured using the Folin–Ciocalteu assay following the procedure of our previous study [22]. An aliquot of 0.2 mL extract was combined with 0.5 mL of 10% Folin–Ciocalteu reagent and 2.25 mL of 7% Na_2_CO_3_, incubated for 90 min, and the absorbance was recorded at 725 nm. TPC values were reported as mg gallic acid equivalents (GAE)/100 g protein (DW). Total flavonoid content (TFC) was quantified as described by Siripan et al. [16]. TFC value was expressed as mg quercetin equivalents (QE)/100 g protein (DW).

#### 2.4.3. Measurement of Antioxidant Activities

Antioxidant activity was determined using the DPPH radical-scavenging assay based on the procedure of Siripan et al. [16], and results were expressed as mg ascorbic acid equivalents (AA)/100 g protein (DW). Ferric reducing antioxidant power (FRAP) was evaluated following the method of Siripan et al. [16], and values were reported as mg Ferrous sulfate (FeSO_4_)/100 g protein DW.

### 2.5. Determination of Protein Content, Gel Electrophoresis SDS-PAGE, and Amino Acid Compositions

#### 2.5.1. Determination of Protein Contents

Crude protein was quantified using the Kjeldahl method with a nitrogen-to-protein conversion factor of 5.95, following Mariotti et al. (2008) [23].

#### 2.5.2. Sodium Dodecyl Sulfate Polyacrylamide Gel Electrophoresis SDS-PAGE

##### Protein Extraction

Rice bran proteins were extracted from Section Rice Bran Protein Extraction following the protocol described by Sari et al. (2019) [24]. Approximately 25 mg of rice bran was homogenized on ice using a pre-chilled extraction buffer (62.5 mM Tris-HCl, pH 6.8; 2.5% SDS; 10% glycerol; and 5% 2-mercaptoethanol). The homogenate was then incubated on a shaker at room temperature for 1 h. Subsequently, the mixture was centrifuged at 15,000 rpm for 15 min at 4 °C. The resulting supernatant was collected and stored at −20 °C until further use.

##### Electrophoresis Analysis

Electrophoresis was performed on polyacrylamide gels, as described by Sari et al. (2019) [24], and there are some minor changes. The resolving gel solution was prepared by combining 3125 µL of 30% acrylamide, 2750 µL of 1 M Tris buffer (pH 8.8), 1505 µL of distilled water, 75 µL of 10% SDS, 75 µL of 10% APS, and 6.25 µL of TEMED. Protein samples were solubilized in an equal volume (1×) of reducing sample buffer containing 1 M Tris-HCl (pH 6.8), 50% glycerol, 10% SDS, 5% β-mercaptoethanol, distilled water, and 1% bromophenol blue. The mixture was heated at 100 °C for 5 min and then immediately cooled on ice until use. Samples were subsequently loaded into the wells of a stacking gel prepared with 0.45 mL of 30% acrylamide, 0.38 mL of 1 M Tris buffer (pH 6.8), 2.11 mL of distilled water, 30 µL of 10% SDS, 30 µL of 10% APS, and 5 µL of TEMED. SDS-PAGE was carried out at 120 V for 90 min, after which the gels were stained with Coomassie Brilliant Blue.

#### 2.5.3. Rice Bran Protein Characterization by FTIR

Functional groups were analyzed using an INVENIO S/LUMOS II FTIR spectrometer (Bruker, Fällanden, Switzerland) in transmission mode from 4000–400 cm^−1^, at a resolution of 4 cm^−1^.

#### 2.5.4. Amino Acid Profiling of Rice Bran Proteins

To remove residual lipids, 1 g of powdered sample was combined with n-hexane (10 mL), stirred for 30 min, and centrifuged. The extraction was repeated three times. Defatted powders were hydrolyzed according to Taesuk et al. (2025) [25]. The hydrolysate was reconstituted (1:10 *w*/*v*) in purified water, centrifuged (12,000 rpm, 10 min), and filtered through a 0.22 µm nylon membrane prior to LC–MS/MS analysis (LCMS-8030, Shimadzu, Kyoto, Japan). Amino acids were analyzed using the method described in Siripan et al. [16].

### 2.6. Data Analysis

All analyses were performed in triplicate. Results are expressed as mean ± standard deviation (SD). Significant differences among treatments were evaluated using one-way ANOVA, followed by Duncan’s multiple range test at a significance level of *p* < 0.05. Statistical analyses were performed using SPSS version 23.0 (SPSS Inc., Chicago, IL, USA) and amino acid taste visualization was generated using OriginPro 2022 (OriginLab, Northampton, MA, USA).

## 3. Results and Discussion

### 3.1. Physicochemical Composition of Rice Bran Protein Extract

The color parameters (CIE L*, a*, and b*) of rice bran protein (RB) and green rice bran protein (GB), as shown in Table 1, varied notably with the extraction method. Enzymatic treatments consistently yielded lower L* values than the non-enzymatic control, indicating darker powders, likely due to pigment release or mild browning during hydrolysis. Among all treatments, PT-AA produced the lowest L* values in both RB and GB. Enzyme-assisted extraction also increased a* and b* values, reflecting enhanced red and yellow hues. PT and PT-AA generated the highest a* values in RB, possibly due to greater phenolic exposure, whereas the increase in b* values, particularly in GB, suggests improved liberation of yellow pigments [26].

The a_w_ value of the samples ranged from 0.34 to 0.53 (as shown in Table 1), remaining well below the microbial growth threshold (a_w_ < 0.60), confirming the microbiological stability of all extracts. As commonly applied in HACCP systems, a_w_ provided useful insight into moisture binding and powder stability [27]. The slightly elevated a_w_ values observed in PT-AA and AA-PT samples may reflect differences in structural compactness and water mobility within the protein matrix.

Moisture content also differed among treatments, as shown in Table 1. PT-AA produced the highest moisture levels in both RB and GB, likely due to the formation of low-molecular-weight peptides with enhanced water-binding capacity. In contrast, AA-treated samples exhibited lower moisture contents, possibly resulting from starch degradation and the formation of a denser matrix. Overall, moisture values ranged narrowly between 1.11% and 1.38%. RB-Control and RB-AA showed the lowest levels (1.11%), indicating more efficient water removal after extraction, likely facilitated by enzyme-mediated structural modification [10].

Overall, enzymatic treatments, particularly PT-AA, altered color attributes, moisture behavior, and powder stability, reflecting greater structural reorganization of the protein matrix and stronger pigment protein interactions. These characteristics are relevant for applications in foods, beverages, and cosmetics, where visual quality and physical stability are essential [17].

### 3.2. Evaluation of Functional Properties of Rice Bran Protein Extracts

#### 3.2.1. Determination of WHC, OHC, and Solubility

Table 2 shows the WHC, OHC, and solubility of rice bran (RB) and green bran (GB) protein extracts obtained from different enzymatic treatments. Significant differences (*p* < 0.05) were observed among treatments and between bran types, indicating that both the extraction method and maturity stage influenced protein functionality.

The sequential AA-PT treatment substantially increased the WHC of GB to 1.85 g/g, more than twice the control value (0.78 g/g), and higher than that of RB under the same conditions. GB-PT-AA also showed the highest OHC (1.61 g/g), significantly exceeding the control (1.34 g/g). OHC reflects a protein’s ability to bind lipids, an important feature for texture, flavor retention, and emulsion stability in fat-rich foods such as sausages, dressings, and plant-based analogs [28]. These improvements suggest that sequential protease and α-amylase treatments effectively exposed hydrophobic residues. Hydrolysis exposes polar groups as well as previously buried hydrophobic residues, effectively opening the protein structure. This structural change enhances the ability of the proteins to interact with water and lipids [29]. The PT and PT-AA-treated fractions showed significantly higher WHC and OHC, particularly in GB, where peptide exposure was more pronounced.

Enzymatic extraction also improved protein solubility. RB-AA-PT exhibited the highest solubility (87.99%), nearly double the control (49.01%). This indicates that enzymatic treatment reduced aggregation, increased surface charge, and promoted better dispersion in aqueous systems. These findings align with Hamada (1999) [30], who reported that protease hydrolysis enhances both solubility and WHC of rice bran protein, and with Grau-Fuentes et al. (2024) [31], who observed significant improvements in WHC, OHC, and solubility following multi-enzyme treatment of defatted rice bran. Proteolysis produced smaller, more charged peptides with reduced aggregation tendency, allowing them to disperse more easily in aqueous systems [32]. This trend was evident in our data, where PT and PT-AA consistently showed the highest solubility in both RB and GB samples (Table 2). These observations align with previous work demonstrating that moderate hydrolysis disrupts hydrophobic interactions and improves solubility. Compared with reports on commercial rice protein isolates, which often show solubility below 20% at neutral pH and WHC around 2–3 g/g protein, the improvements observed here highlight the effectiveness of enzymatic treatments in enhancing hydration and dispersibility [33].

#### 3.2.2. Foaming Capacity and Stability

The foaming capacity (FC) and foaming stability (FS) of rice bran protein extracts are summarized in Table 2. In fully ripe rice bran (RB), PT-AA produced the highest FC (72%) with moderate FS (52%), whereas AA resulted in the lowest FC (65%) and FS (43%). Sequential treatments (AA-PT and PT-AA) significantly improved foaming relative to the control, indicating that combined hydrolysis generates more flexible protein structures capable of stabilizing air–water interfaces [12]. In green bran (GB), AA-PT yielded the highest FC (97%) and FS (65%), demonstrating superior foam-forming ability and structural stability. Although PT-AA also produced high FC (84%), its FS (52%) was comparatively lower. These patterns are consistent with findings from Yang et al. (2023) [34] and Braspaiboon et al. (2020) [35], who reported that moderate proteolysis enhances foaming and emulsifying properties in cereal and legume proteins by producing peptides with increased mobility and interfacial activity. The strong foaming performance of GB samples suggests that proteins from green rice bran possess inherently greater structural flexibility, which may be advantageous for aerated food systems. The presence of low to medium-molecular-weight peptides increases molecular mobility and promotes faster adsorption at the air–water interface [36]. This explains why PT-AA presented the best foaming performance in RB, while AA-PT was superior in GB. These results emphasize that proteolysis improves flexibility and interfacial film formation, key factors in foam generation.

However, the reduced FS observed in certain PT–AA extracts indicates that excessive hydrolysis may generate peptides too small to form stable interfacial films, emphasizing the need for controlled proteolysis [37]. These trends align with values reported for commercial rice protein isolates and other plant proteins. For instance, acetylation at 0.4 mL/g has been shown to increase WHC relative to the control (2.07 g/g), while a higher dosage (2.0 mL/g) nearly doubled WHC to 3.76 g/g [17]. Comparatively, commercial plant proteins further highlight these advantages. Kidney bean flour typically exhibits OHC values of 2.2–2.3 g/g [38], whereas oats and wheat commonly present WHC values around 1.5 g/g. Protein solubility in wheat, oats, and peas generally ranges from 4–10%, reflecting the limited aqueous dispersibility of many plant protein isolates. Foaming characteristics also vary widely across plant sources; wheat proteins, for example, may exhibit FC values approaching 98%, although FS does not necessarily correspond to FC [33]. Collectively, these comparisons underscore the functional enhancements achieved through enzymatic modification, which markedly improved solubility, hydration capacity, and foaming behavior in both RB and GB extracts.

### 3.3. Characterization of Bioactive Compounds and Antioxidant Activities in Rice Bran Protein Extract

Table 3 presents the total phenolic content (TPC), total flavonoid content (TFC), and antioxidant activities (DPPH and FRAP) of protein extracts from rice bran (RB) and green bran (GB) subjected to different enzymatic treatments. Enzyme type and sequence significantly affected phenolic release and antioxidant potential.

In RB, the PT-AA treatment showed the highest DPPH scavenging activity (89 mg AA/100 g DW), nearly three times higher than the control (32 mg AA/100 g DW), indicating strong synergistic effects of sequential hydrolysis on bioactive peptide formation. In GB, protease treatment (PT) produced the highest FRAP value (2472 mg FeSO_4_/100 g DW), over fivefold greater than the control (473 mg FeSO_4_/100 g DW), reflecting increased liberation of reducing peptides. GB-AA also displayed the highest TFC (1069 mg QE/100 g DW), more than fivefold higher than the control, suggesting that α-amylase effectively released bound flavonoids. Applying protease after α-amylase (AA-PT) yielded the greatest improvements in GB. This suggests that initial starch disruption allows protease to access storage proteins more effectively. These results are in agreement with Andriani et al. (2022) [26], who reported that enzymatic hydrolysis efficiently releases cell wall-bound phenolics and flavonoids, and with Thamnarathip et al. (2016) [39], who observed significant increases in TPC and antioxidant activity in Riceberry bran following enzyme-assisted hydrolysis. Similarly, Kim and Lim (2016) [40] demonstrated that α-amylase enhances phenolic extractability and radical-scavenging activity.

Overall, the findings demonstrate that both the enzyme type and the sequence of application significantly influence the bioactive and antioxidant profiles of RB and GB protein extracts. Treatments such as RB-PT-AA, GB-PT, and GB-AA produced the most substantial enhancements, underscoring enzyme-assisted extraction as an effective strategy to improve the nutritional and functional attributes of rice bran proteins.

### 3.4. Assessment of Protein Content and Amino Acid Profiles in Rice Bran Protein Extract

#### 3.4.1. Rice Bran Protein Content

Enzymatic extraction markedly enhanced protein enrichment and recovery compared with the non-enzymatic control. In fully ripe bran (RB), protease treatment (PT) produced the highest protein content (23.41%), more than threefold greater than the control (7.53%). Sequential α-amylase–protease extraction (AA-PT) resulted in the highest recovery (8.48%), nearly four times that of the control (2.15%). In green bran (GB), PT again yielded the highest protein content (28.53%) and recovery (8.58%), significantly exceeding the control values (7.88% and 2.96%, respectively). Traditional alkaline extraction of rice flour can generate protein concentrates with up to 80% protein, but such methods may leave undesirable chemical residues if raw materials are not carefully managed [9]. Enzyme-assisted extraction offers a safer alternative. Scarabattoli et al. (2023) [41] reported a comparable 105% increase in protein enrichment from rice bran following carbohydrase application. Likewise, Tang et al. (2003) [12] observed that physical pretreatments including freeze–thaw, sonication, and high-speed mixing improved protein yield by approximately 5% relative to non-enzymatic methods, and Lasrichan et al. (2024) [42] emphasized the importance of raw material preparation for optimizing extraction efficiency.

Residue recovery ranged between 70–75%. In RB, the control retained the highest residue (74%), whereas PT-AA produced the lowest (70%), indicating more effective solubilization of bran components. GB samples showed slightly higher residues overall, suggesting maturity-dependent differences in how enzymes interact with the matrix. Statistical analysis (*p* < 0.05) confirmed significant effects of both enzyme sequence and maturity stage.

Overall, protease-based extraction (PT) provided the best balance between protein yield and enrichment. Sequential enzyme treatments may offer additional benefits but require optimization to prevent efficiency losses. Given the intrinsic protein content of rice bran (~9.8%), comparable to the 7.2–11.5% range reported for rice grains [43], protease-assisted extraction, particularly for green rice bran, represents a scalable and sustainable approach for producing high-quality protein suitable for food and nutraceutical applications.

#### 3.4.2. SDS-PAGE

**Figure 2:**
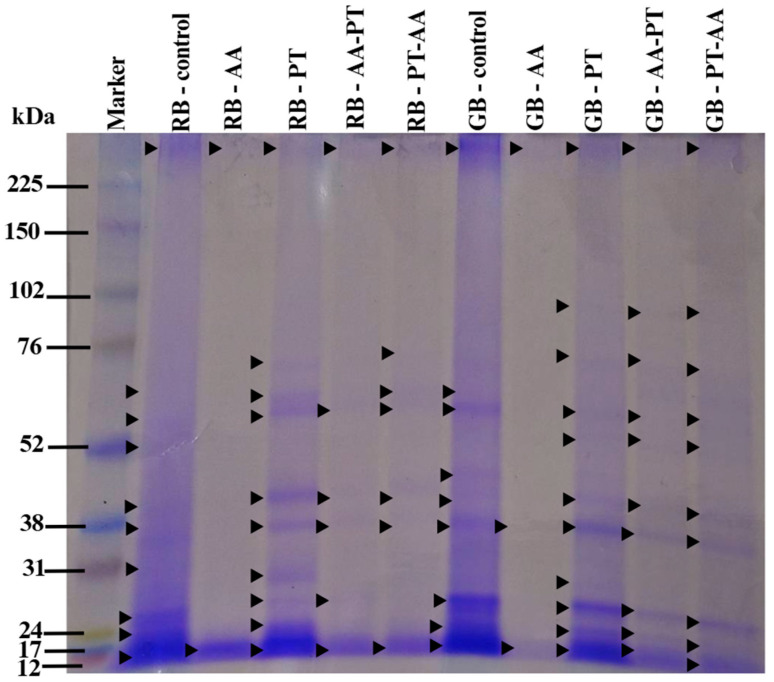
SDS-PAGE profiles of rice bran protein extracts obtained from different enzymatic treatments. Lane M: molecular weight marker (10–225 kDa). RB and GB denote protein extracts from fully ripe and green KDML 105 rice bran, respectively. Treatments include a non-enzymatic control; single-enzyme extractions using α-amylase (AA) and protease (PT); and sequential extractions (AA-PT and PT-AA).

SDS-PAGE analysis revealed clear differences in protein profiles among treatments (Figure 2). The RB-AA fraction displayed only two prominent low-molecular-weight bands (~12–17 kDa), whereas the control and protease-treated samples exhibited multiple bands ranging from 12 to >100 kDa. The marked reduction in band diversity in RB-AA indicates that α-amylase disrupted the starch–protein matrix, causing extensive protein solubilization and loss during centrifugation, resulting in low protein recovery (Table 4) [12].

The remaining bands in RB-AA (~12–17 kDa) correspond to rice prolamins, which typically appear at 10–16 kDa [44,45]. Previous reports show that rice prolamins, particularly the 16.6 kDa cysteine-rich form, are highly hydrophobic and enriched in leucine, proline, glutamic acid, glutamine, and methionine, while being low in lysine and sulfur-containing amino acids [46,47]. This agrees with the amino acid profile of RB-AA (Table 4), supporting the interpretation that α-amylase preferentially enriches prolamin-like fractions while reducing glutelin and albumin/globulin components.

GB-AA also exhibited reduced band intensity; however, its WHC and OHC remained comparable to the control. This suggests that the more porous structure of green bran may better retain functional properties following starch hydrolysis. By contrast, RB showed greater losses in functionality due to extensive disruption of starch protein associations.

SDS-PAGE followed by Coomassie Brilliant Blue (CBB) staining revealed up to 11 distinct protein bands (Figure 2). Approximately 10 protein bands were observed in both RB-Control and GB-Control samples, with molecular weights ranging from 12 to 102 kDa. Ten bands were detected in RB-PT samples (12–102 kDa and protein band above the marker weighs more than 225 kDa above the Marker Bar), while 11 bands were observed in GB-PT samples (12–225 kDa), which were similar to the profiles of GB-AA-PT and GB-PT-AA samples. In contrast, RB-AA and GB-AA samples exhibited low protein content and predominantly lower-molecular-weight proteins. It can be observed from the intensity of the colored bands on SDS-PAGE. This may be attributed to the action of α-amylase, which hydrolyzes starch into sugars that can facilitate protein extraction.

Samples treated with protease displayed weaker bands together with the presence of smaller peptide fragments, indicating that partial hydrolysis had occurred. The FTIR spectra also showed shifts in the amide I and amide II regions [48], reflecting increases in beta sheet and random coil structures as well as partial unfolding. These observations were consistent with the changes detected in amino acid composition [49]. Overall, the structural alterations help explain the improvements in functional properties reported in this study [49,50].

In a related study, Phonsakhan & Kong-Ngern (2015) [51] identified multiple protein spots in black glutinous rice leaves using 2D-PAGE and LC–MS/MS, with several proteins being differentially expressed. Zhang et al. (2013) [52] reported a 54 kDa chloroplast-associated band in Japonica rice, while Santos et al. (2013) [53] predicted three major proteins in *Oryza glumaepatula*: glutelin (34–36 kDa), albumin (15–25 kDa), and prolamin (15–18 kDa). Distinct protein patterns at various developmental stages have been linked to metabolic processes such as pigmentation, storage protein accumulation, and metabolite biosynthesis. These characteristic banding patterns can serve as biochemical markers for rice species differentiation, and previous research has demonstrated that SDS-PAGE profiles can effectively reflect genetic diversity [24].

#### 3.4.3. Characterization of Rice Bran Protein Using FTIR Spectroscopy

**Figure 3:**
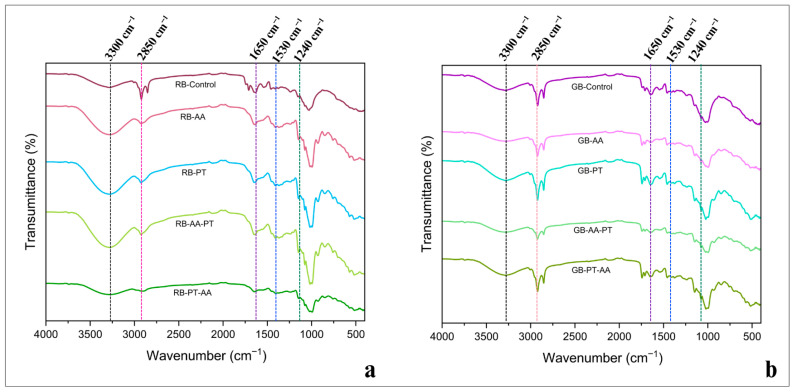
Characterization of Rice Bran Protein Using FTIR Spectroscopy with different extraction methods. Appearance of (**a**) is the FTIR image of RB rice bran protein sample, and (**b**) is the FTIR image of GB rice bran protein sample. RB and GB denote protein extracts from fully ripe and green KDML 105 rice bran, respectively. Treatments include a non-enzymatic control; single-enzyme extractions using α-amylase (AA) and protease (PT); and sequential extractions (AA-PT and PT-AA).

Changes in the secondary structure of enzymatically hydrolyzed rice bran protein extracts were evaluated using FTIR spectroscopy. Shifts in the amide I and II bands, typically associated with peptide backbone cleavage and increased exposure of hydrophilic and hydrophobic groups, confirmed the structural alterations induced by enzymatic treatments [50]. FTIR analysis of the GB and RB protein extracts (Figure 3a,b) consistently exhibited characteristic protein bands, including Amide I (≈1650 cm^−1^), Amide II (≈1530 cm^−1^), and Amide III (≈1240 cm^−1^) bands, indicating that the peptide backbone was largely preserved across treatments [54]. Meanwhile, C–H stretching bands at 2850–2950 cm^−1^, corresponding to –CH_2_– and –CH_3_ groups, decreased in intensity after enzymatic hydrolysis, suggesting partial removal of associated lipids and structural reorganization. Distinct differences between GB and RB samples were observed. GB displayed clearer absorption at 1240, 1550, and 1650 cm^−1^, reflecting well-defined protein functional groups. A pronounced broad band near 3300 cm^−1^ particularly in PT, AA-PT, and PT-AA treatments indicated enhanced hydrogen bonding interactions associated with protein unfolding and disruption of lipid–protein complexes. Reduction in peak intensities, especially in PT and PT-AA, further suggested loosening and rearrangement of the protein matrix. In RB samples, enzymatic extraction most notably AA-PT and PT-AA induced shifts and increased transmittance in the amide I and II regions, implying partial unfolding and increased exposure of polar amino acids [55]. These spectral shifts correspond to a decrease in α-helix structures (≈1650–1658 cm^−1^) and a relative increase in β-sheet or random coil conformations (≈1660–1670 cm^−1^) [56,57]. The more prominent structural features observed in GB may be attributed to its higher inherent moisture content, which improves enzyme accessibility and promotes more efficient hydrolytic cleavage [49].

The FTIR findings corroborate the SDS-PAGE and amino acid composition results. The appearance of low-molecular-weight peptides and reduced band intensity in protease-treated fractions align with the observed increase in β-sheet/random coil structures [48,58]. Furthermore, the exposure of hydrophobic and charged amino acids following protease treatment parallels the intensity changes in amide I–II regions [59]. Altogether, these results confirm that enzymatic extraction induces secondary structural modifications that underpin improvements in protein solubility, foaming capacity, and antioxidant activity [50].

#### 3.4.4. Composition and Content of Amino Acids

Amino acids determine both the nutritional quality and sensory attributes of proteins, contributing to sweetness, bitterness, and umami characteristics. They also serve as key metabolic intermediates in plants and are essential for the nutritional value of cereal grains [60,61,62].

Enzymatic treatments markedly influenced the amino acid composition. In RB, the RB-AA fraction exhibited the highest total amino acid content, followed by RB-PT and RB-PT-AA. Notably, leucine in RB-PT-AA was 6.49-fold higher than in the control. In GB, GB-PT-AA showed the highest total amino acid content, with leucine levels 21.26-fold greater than the GB-control. Non-essential amino acids were also significantly increased, particularly in GB-PT-AA (~4.18-fold) and GB-PT (~3.76-fold), confirming that protease alone or in combination with α-amylase enhances protein extraction and improves nutritional quality.

**Figure 4:**
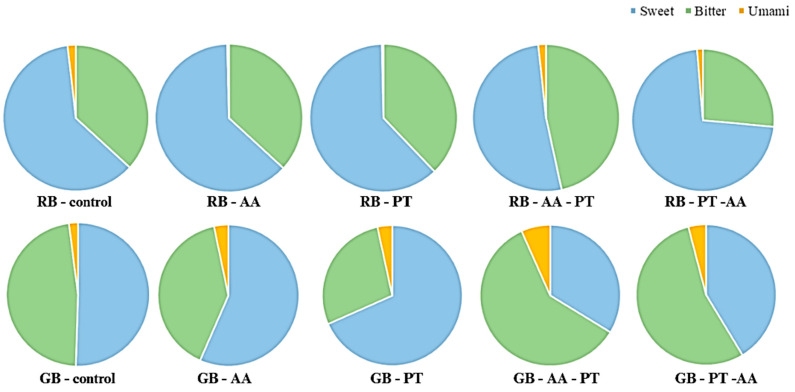
The taste of the amino acid of rice bran protein extracts obtained from different enzymatic treatments. Values are expressed as mean ± SD (n = 3). Different letters indicate significant differences among treatments (*p* < 0.05). RB and GB denote protein extracts from fully ripe and green KDML 105 rice bran, respectively. Treatments include a non-enzymatic control; single-enzyme extractions using α-amylase (AA) and protease (PT); and sequential extractions (AA-PT and PT-AA).

Taste-active amino acids varied distinctly among extraction methods (Figure 4). Sweetness-related amino acids (alanine, glycine, serine) increased with α-amylase treatment, while umami-rich amino acids (glutamic and aspartic acids) were elevated by protease, especially in PT and PT-AA samples. Bitter amino acids (arginine, phenylalanine, histidine, valine, tryptophan, isoleucine, leucine) were also enhanced through proteolysis [63,64]. Protease treatment substantially increases in amino acids linked to umami and bitterness, with PT markedly elevating glutamic acid, a key umami compound [65]. Conversely, α-amylase treatment increased the levels of sweet-tasting amino acids, consistent with its role in contributing to milder flavor attributes [66]. Sequential enzyme applications further diversified the amino acid profile, with PT and PT-AA producing the greatest enhancement of umami-related amino acids, whereas AA-based treatments improved sweetness. Overall, GB extracts exhibited stronger responses than RB, reflecting the greater protein extractability of green rice bran.

Enzymatic extraction, particularly treatments involving protease and the sequential application of protease followed by α-amylase, resulted in clear and measurable structural alterations in rice bran proteins [11]. Evidence from SDS-PAGE showed that enzyme-assisted extractions yielded higher protein recovery, reflected by intensified bands associated with low-molecular-weight peptides [49]. These observations indicate enhanced proteolysis and the release of soluble peptide fractions. Complementary FTIR analysis further confirmed these structural changes. Enzyme-treated samples displayed more pronounced β-sheet features and clear Amide I (≈1650 cm^−1^), Amide II (≈1530 cm^−1^), and Amide III (≈1240 cm^−1^) peaks, along with an overall increase in amino acid availability [42]. Together, these findings demonstrate partial hydrolysis and modifications to the secondary structural organization of the proteins.

The Degree of Hydrolysis (DH) is commonly used as an indicator of the extent of proteolysis. However, Wouters et al. (2016) [67] noted that DH alone does not adequately describe the structural characteristics of protein hydrolysates. Samples with similar DH values may still exhibit distinct structural features and functional behaviors, depending on the specificity of the enzymes used and the protein source.

In addition, previous studies employing DSC to assess thermal transitions have demonstrated that enzymatic hydrolysis can alter the thermal stability of proteins. Dent et al. (2023) [68] reported that hydrolysis with Alcalase reduced the maximum denaturation temperatures of soy and chickpea proteins to 77 °C and 86 °C, respectively, whereas treatment with Flavourzyme increased these temperatures to 93 °C and 92 °C. These findings further support the role of enzymatic modification in influencing protein thermal behavior through changes in molecular conformation and peptide interactions.

Overall, this study demonstrates that sequential enzyme-assisted extraction induces partial hydrolysis and significantly alters the secondary structure of rice bran proteins, resulting in marked improvements in their functional properties. The combined structural and functional evidence confirms that enzymatic extraction is an effective strategy for enhancing the quality and application potential of rice bran proteins in food systems.

## 4. Conclusions

Enzymatic extraction, particularly protease (PT) and the sequential protease followed by α-amylase (PT-AA) treatments, improved protein recovery and enhanced key functional properties of rice bran proteins. Green rice bran showed greater responsiveness to enzymatic hydrolysis, with higher solubility, hydration capacity, and foaming performance. These functional improvements were supported by structural changes observed in SDS-PAGE and FTIR analyses. The amino acid composition further indicated that the extracted proteins retained a balanced profile, reinforcing their nutritional potential. While these findings demonstrate clear functional benefits under controlled laboratory conditions, their performance under real food-processing environments remains unverified. Future studies should therefore assess thermal and mechanical stability, evaluate functionality in model food systems, compare performance with commercial protein isolates, and examine process scalability. Investigation of amino acid stability during processing and storage is also recommended. Thus, future work should systematically evaluate how varying incubation time, enzyme concentrations, temperature, and pH interact with enzyme sequence to further optimize protein extraction efficiency and functional properties.

## Figures and Tables

**Table 1:** Physicochemical assessment of rice bran protein obtained from different extraction methods.

Treatment		Color			a_W_^NS^	Moisture Content (% DW)
		L*	a*	b*		
RB	control	72.88 ± 0.57 ^a^	1.63 ± 0.51 ^d^	8.32 ± 0.57 ^g^	0.53 ± 0.01	1.11 ± 0.02 ^g^
	AA	61.55 ± 0.05 ^c^	2.75 ± 0.01 ^c^	13.24 ± 0.01 ^e^	0.50 ± 0.10	1.11 ± 0.01 ^g^
	PT	59.46 ± 0.05 ^e^	3.79 ± 0.01 ^a^	14.65 ± 0.01 ^d^	0.49 ± 0.10	1.21 ± 0.01 ^d^
	AA-PT	61.45 ± 0.05 ^c^	2.75 ± 0.01 ^c^	12.89 ± 0.05 ^f^	0.45 ± 0.10	1.18 ± 0.01 ^e^
	PT-AA	59.81 ± 0.05 ^e^	3.94 ± 0.01 ^a^	14.87 ± 0.01 ^d^	0.47 ± 0.01	1.38 ± 0.02 ^a^
GB	control	60.68 ± 0.01 ^d^	3.33 ± 0.03 ^b^	15.37 ± 0.04 ^c^	0.47 ± 0.10	1.24 ± 0.02 ^c^
	AA	53.39 ± 0.13 ^f^	3.81 ± 0.07 ^a^	20.17 ± 0.04 ^a^	0.50 ± 0.20	1.12 ± 0.01 ^g^
	PT	59.58 ± 0.25 ^e^	0.51 ± 0.01 ^e^	16.77 ± 0.09 ^b^	0.53 ± 0.20	1.32 ± 0.01 ^b^
	AA-PT	61.46 ± 0.01 ^c^	2.77 ± 0.02 ^c^	12.9 ± 0.01 ^f^	0.49 ± 0.20	1.21 ± 0.01 ^d^
	PT-AA	67.55 ± 0.02 ^b^	1.35 ± 0.05 ^d^	20.31 ± 0.02 ^a^	0.48 ± 0.50	1.14 ± 0.02 ^f^

The values are mean ± SD (n = 3). Means with different letters (^a,b,c,…^) are significantly different at *p* < 0.05 within the same column in the parameter. within the same column for each parameter. RB and GB denote protein extracts from fully ripe and green KDML 105 rice bran, respectively. Treatments include a non-enzymatic control; single-enzyme extractions using α-amylase (AA) and protease (PT); and sequential extractions (AA-PT and PT-AA). ^NS^ = not significantly different.

**Table 2:** Functional characteristics of rice protein produced by different extraction methods.

Treatment		WHC (g/g)	OHC (g/g)	Solubility Content (%)	Foaming Capacity (%)	Foaming Stability (%)
RB	control	0.46 ± 0.01 ^ef^	1.25 ± 0.01 ^e^	49.01 ± 1.54 ^f^	24.36 ± 0.05 ^h^	34.80 ± 0.08 ^j^
	AA	0.31 ± 0.01 ^g^	0.91 ± 0.01 ^g^	69.60 ± 2.03 ^e^	65.40 ± 0.43 ^e^	43.60 ± 0.29 ^h^
	PT	0.49 ± 0.01 ^e^	1.22 ± 0.01 ^e^	80.94 ± 0.53 ^b^	72.83 ± 0.55 ^d^	52.02 ± 0.39 ^e^
	AA-PT	0.68 ± 0.01 ^c^	1.15 ± 0.03 ^f^	87.99 ± 1.19 ^a^	58.30 ± 0.26 ^f^	36.43 ± 0.16 ^i^
	PT-AA	0.41 ± 0.01 ^f^	1.46 ± 0.01 ^c^	87.82 ± 0.73 ^a^	73.13 ± 0.05 ^d^	48.75 ± 0.03 ^f^
GB	control	0.78 ± 0.01 ^b^	1.34 ± 0.01 ^d^	46.90 ± 0.67 ^g^	44.16 ± 0.05 ^g^	63.09 ± 0.08 ^b^
	AA	0.80 ± 0.02 ^b^	1.68 ± 0.01 ^a^	76.28 ± 0.30 ^c^	85.16 ± 0.05 ^b^	56.77 ± 0.03 ^c^
	PT	0.68 ± 0.11 ^c^	1.47 ± 0.07 ^c^	72.08 ± 0.90 ^d^	79.36 ± 0.05 ^c^	46.68 ± 0.03 ^g^
	AA-PT	1.85 ± 0.02 ^a^	1.56 ± 0.03 ^b^	78.11 ± 0.45 ^c^	97.58 ± 0.17 ^a^	65.05 ± 0.11 ^a^
	PT-AA	0.56 ± 0.01 ^d^	1.61 ± 0.05 ^b^	70.25 ± 1.14 ^de^	84.36 ± 1.61 ^b^	52.72 ± 1.01 ^d^

The values are mean ± SD (n = 3). Means with different letters (^a,b,c,…^) are significantly different at *p* < 0.05 within the same column in the parameter. within the same column for each parameter. RB and GB denote protein extracts from fully ripe and green KDML 105 rice bran, respectively. Treatments include a non-enzymatic control; single-enzyme extractions using α-amylase (AA) and protease (PT); and sequential extractions (AA-PT and PT-AA).

**Table 3:** Characterization of bioactive compounds and antioxidant activity in rice bran protein extract.

Treatment		TPC (mg GAE/100 g Protein DW)	TFC (mg QE/100 g Protein DW)	DPPH (mg AA/100 g Protein DW)	FRAP (mg FeSO_4_/100 g Protein DW)
RB	control	17.63 ± 1.09 ^d^	159.69 ± 1.30 ^h^	32.41 ± 1.12 ^g^	241.70 ± 1.66 ^g^
	AA	16.04 ± 0.43 ^e^	397.38 ± 1.10 ^c^	76.22 ± 0.22 ^c^	299.94 ± 1.44 ^f^
	PT	14.74 ± 0.43 ^f^	357.38 ± 1.80 ^d^	72.45 ± 0.34 ^de^	396.41 ± 1.69 ^e^
	AA-PT	15.32 ± 0.50 ^ef^	200.46 ± 1.52 ^g^	56.27 ± 0.13 ^f^	394.05 ± 1.76 ^e^
	PT-AA	17.20 ± 0.25 ^d^	192.92 ± 0.01 ^g^	89.92 ± 0.56 ^a^	285.23 ± 1.03 ^f^
GB	control	23.66 ± 0.30 ^c^	197.69 ± 1.38 ^g^	74.94 ± 1.69 ^cd^	473.47 ± 1.44 ^d^
	AA	26.49 ± 0.17 ^b^	1069.69 ± 1.66 ^a^	73.80 ± 0.24 ^cd^	639.94 ± 1.68 ^b^
	PT	28.85 ± 0.10 ^a^	318.92 ± 0.01 ^e^	70.02 ± 1.90 ^e^	2472.88 ± 1.66 ^a^
	AA-PT	26.39 ± 0.15 ^b^	221.23 ± 1.80 ^f^	88.91 ± 0.86 ^a^	517.58 ± 1.29 ^c^
	PT-AA	25.78 ± 0.13 ^b^	434.30 ± 1.21 ^b^	81.93 ± 0.53 ^b^	647.00 ± 1.75 ^b^

The values are mean ± SD (n = 3). Means with different letters (^a,b,c,…^) are significantly different at *p* < 0.05 within the same column in the parameter. RB and GB denote protein extracts from fully ripe and green KDML 105 rice bran, respectively. Treatments include a non-enzymatic control; single-enzyme extractions using α-amylase (AA) and protease (PT); and sequential extractions (AA-PT and PT-AA).

**Table 4:** Amino acid composition and content of rice bran protein obtained through different extraction methods.

Protein and Amino Acid Content (mg/g DW)	RB-Control	RB-AA	RB-PT	RB-AA-PT	RB-PT-AA	GB-Control	GB-AA	GB-PT	GB-AA-PT	GB-PT-AA
Protein (%)	7.53 ± 3.09 ^f^	11.51 ± 0.02 ^e^	23.41 ± 0.52 ^b^	14.58 ± 0.90 ^d^	18.95 ± 0.26 ^c^	7.88 ± 0.98 ^f^	10.42 ± 0.01 ^e^	28.53 ± 0.63 ^a^	17.58 ± 0.34 ^c^	19.32 ± 0.53 ^c^
% Yield	2.15 ± 0.07 ^g^	6.87 ± 0.01 ^c^	8.48 ± 0.01 ^a^	6.58 ± 0.01 ^d^	5.11 ± 0.01 ^e^	2.96 ± 0.01 ^f^	6.87 ± 0.01 ^c^	8.58 ± 0.01 ^a^	7.48 ± 0.01 ^b^	7.35 ± 0.48 ^b^
Residue from extraction (% Yield)	74.69 ± 0.62 ^abc^	74.63 ± 0.17 ^bc^	73.04 ± 1.02 ^d^	71.59 ± 0.06 ^e^	70.44 ± 0.30 ^f^	75.50 ± 0.68 ^a^	74.44 ± 0.04 ^bc^	74.8 ± 0.23 ^ab^	74.62 ± 0.16 ^bc^	73.87 ± 0.28 ^d^
Essential amino acids (EAAs)										
Arginine	2.82 ± 0.01 ^e^	2.98 ± 0.00 ^de^	2.97 ± 0.01 ^ed^	2.77 ± 0.01 ^e^	2.89 ± 0.00 ^e^	3.37 ± 0.02 ^bc^	3.46 ± 0.24 ^b^	3.17 ± 0.01 ^cd^	3.42 ± 0.08 ^bc^	4.64 ± 0.40 ^a^
Histidine	2.03 ± 0.00 ^d^	2.14 ± 0.06 ^b^	2.13 ± 0.01 ^b^	2.01 ± 0.00 ^d^	2.07 ± 0.00 ^c^	2.13 ± 0.02 ^b^	2.07 ± 0.04 ^c^	2.11 ± 0.01 ^b^	2.28 ± 0.01 ^a^	2.26 ± 0.02 ^a^
Isoleucine	2.91 ± 2.13 ^de^	10.22 ± 0.92 ^a^	7.52 ± 1.33 ^b^	0.60 ± 0.43 ^f^	1.23 ± 0.15 ^f^	1.71 ± 0.01 ^ef^	0.48 ± 0.12 ^f^	4.04 ± 0.39 ^cd^	1.87 ± 0.13 ^ef^	5.36 ± 0.16 ^c^
Leucine	2.04 ± 0.22 ^de^	11.72 ± 0.24 ^bc^	9.85 ± 1.44 ^c^	1.55 ± 1.37 ^de^	13.25 ± 4.30 ^b^	0.72 ± 0.02 ^e^	0.25 ± 0.01 ^e^	4.34 ± 0.32 ^d^	4.36 ± 0.43 ^d^	15.31 ± 1.08 ^a^
Lysine	0.65 ± 0.03 ^cd^	1.32 ± 0.10 ^a^	0.91 ± 0.11 ^bc^	0.56 ± 0.04 ^de^	0.35 ± 0.01 ^e^	0.68 ± 0.02 ^cd^	0.99 ± 0.45 ^b^	0.55 ± 0.02 ^de^	0.94 ± 0.01 ^bc^	1.03 ± 0.15 ^b^
Methionine	0.16 ± 0.00 ^c^	4.57 ± 0.41 ^a^	4.87 ± 1.06 ^a^	1.15 ± 0.47 ^b^	0.63 ± 0.15 ^bc^	0.34 ± 0.04 ^bc^	0.35 ± 0.04 ^bc^	0.69 ± 0.32 ^bc^	0.53 ± 0.46 ^bc^	0.25 ± 0.01 ^c^
Phenylalanine	4.57 ± 2.06 ^b^	11.73 ± 2.56 ^a^	11.08 ± 1.59 ^a^	2.82 ± 1.17 ^bcd^	1.47 ± 0.37 ^cd^	0.56 ± 0.12 ^d^	1.70 ± 0.07 ^cd^	4.07 ± 2.52 ^bc^	1.26 ± 0.11 ^d^	1.80 ± 0.17 ^cd^
Threonine	0.11 ± 0.01 ^d^	0.32 ± 0.01 ^c^	0.30 ± 0.01 ^c^	0.13 ± 0.00 ^d^	0.09 ± 0.01 ^d^	0.10 ± 0.01 ^d^	0.12 ± 0.01 ^d^	1.85 ± 0.15 ^a^	1.11 ± 0.07 ^b^	1.19 ± 0.19 ^b^
Tryptophan	NS	NS	NS	NS	NS	NS	NS	NS	NS	NS
Valine	0.04 ± 0.01 ^d^	1.47 ± 0.09 ^a^	1.52 ± 0.21 ^a^	0.42 ± 0.13 ^b^	0.21 ± 0.06 ^bcd^	0.06 ± 0.02 ^d^	0.28 ± 0.14 ^bcd^	0.15 ± 0.00 ^cd^	0.38 ± 0.24 ^bc^	0.22 ± 0.13 ^bcd^
ƩEAAs	15.37 ± 0.34 ^f^	46.5 ± 3.84 ^a^	41.18 ± 5.75 ^b^	12.02 ± 2.78 ^fg^	22.22 ± 5.06 ^d^	9.72 ± 0.05 ^fg^	9.74 ± 0.70 ^fg^	20.98 ± 1.58 ^de^	16.16 ± 1.03 ^ef^	32.11 ± 1.42 ^c^
Non-Essential amino acids (NEAAs)										
Alanine	2.79 ± 0.01 ^de^	21.59 ± 0.73 ^a^	19.83 ± 0.75 ^b^	3.44 ± 0.83 ^d^	2.41 ± 0.36 ^e^	0.55 ± 0.024 ^f^	0.82 ± 0.39 ^f^	0.58 ± 0.02 ^f^	3.69 ± 0.46 ^d^	5.05 ± 0.91 ^c^
Asparagine	0.14 ± 0.00 ^d^	0.14 ± 0.01 ^cd^	0.15 ± 0.00 ^cd^	0.14 ± 0.00 ^d^	0.15 ± 0.00 ^cd^	0.14 ± 0.00 ^d^	0.14 ± 0.01 ^d^	0.27 ± 0.01 ^a^	0.15 ± 0.01 ^bc^	0.16 ± 0.05 ^b^
Aspartic acid	0.25 ± 0.02 ^e^	0.16 ± 0.00 ^e^	0.14 ± 0.07 ^e^	0.29 ± 0.00 ^e^	0.35 ± 0.00 ^e^	1.47 ± 0.19 ^cd^	3.41 ± 0.03 ^a^	2.92 ± 0.03 ^b^	1.28 ± 0.42 ^d^	1.69 ± 0.47 ^c^
Cysteine	2.71 ± 0.15 ^ef^	2.27 ± 0.03 ^f^	3.54 ± 0.01 ^cd^	4.29 ± 0.15 ^b^	4.45 ± 0.56 ^b^	3.17 ± 0.28 ^de^	2.23 ± 0.05 ^f^	3.94 ± 0.02 ^bc^	5.05 ± 0.2 ^a^	5.14 ± 0.72 ^a^
Glutamine	1.47 ± 0.31 ^b^	0.98 ± 0.09 ^b^	0.81 ± 0.06 ^b^	0.50 ± 0.03 ^b^	0.42 ± 0.01 ^b^	0.56 ± 0.02 ^b^	0.96 ± 0.43 ^b^	12.38 ± 1.19 ^a^	1.45 ± 0.61 ^b^	12.37 ± 1.14 ^a^
Glutamic acid	0.31 ± 0.01 ^b^	0.26 ± 0.01 ^b^	0.22 ± 0.01 ^b^	0.29 ± 0.16 ^b^	0.25 ± 0.15 ^b^	0.17 ± 0.01 ^b^	0.35 ± 0.14 ^b^	2.05 ± 0.24 ^a^	2.56 ± 1.42 ^a^	3.14 ± 1.92 ^a^
Glycine	0.66 ± 0.32 ^f^	10.12 ± 0.73 ^b^	10.7 ± 0.31 ^b^	3.53 ± 0.43 ^d^	1.25 ± 0.03 ^ef^	2.16 ± 0.19 ^e^	0.76 ± 0.35 ^f^	25.25 ± 0.12 ^a^	1.87 ± 0.08 ^e^	8.29 ± 1.52 ^c^
Proline	0.22 ± 0.01 ^d^	2.54 ± 0.06 ^a^	2.79 ± 0.73 ^a^	0.75 ± 0.2 ^bc^	0.43 ± 0.07 ^cd^	0.21 ± 0.01 ^d^	1.08 ± 0.37 ^b^	1.13 ± 0.16 ^b^	0.31 ± 0.01 ^cd^	0.36 ± 0.02 ^cd^
Serine	0.40 ± 0.01 ^d^	0.33 ± 0.03 ^de^	0.32 ± 0.01 ^de^	0.23 ± 0.00 ^fg^	0.20 ± 0.00 ^g^	0.18 ± 0.01 ^g^	0.29 ± 0.04 ^ef^	1.93 ± 0.02 ^a^	0.9 ± 0.08 ^b^	0.74 ± 0.1 ^c^
Tyrosine	0.68 ± 0.02 ^de^	5.09 ± 0.58 ^c^	6.24 ± 1.34 ^c^	1.23 ± 0.91 ^de^	0.67 ± 0.2 ^de^	1.28 ± 0.06 ^de^	0.07 ± 0.06 ^e^	3.36 ± 0.49 ^cd^	9.84 ± 4.69 ^b^	14.24 ± 0.92 ^a^
ƩNEAAs	9.66 ± 0.44 ^e^	43.50 ± 0.64 ^b^	44.76 ± 2.67 ^b^	14.69 ± 1.25 ^d^	10.59 ± 0.28 ^e^	9.92 ± 0.23 ^e^	10.17 ± 1.43 ^e^	53.83 ± 3.31 ^a^	27.13 ± 5.83 ^c^	51.2 ± 0.56 ^a^
ƩAAs	25.04 ± 0.79 ^e^	90.01 ± 4.48 ^a^	85.95 ± 8.42 ^a^	26.73 ± 4.03 ^de^	32.83 ± 5.35 ^d^	19.65 ± 0.28 ^e^	19.92 ± 2.14 ^e^	74.83 ± 4.90 ^b^	43.3 ± 4.79 ^c^	83.33 ± 0.86 ^a^
ƩEAAs/ƩAAs (%)	61.42	51.66	47.91	44.98	67.71	49.44	48.97	28.04	37.34	38.52
ƩNEAAs/ƩAAs (%)	38.58	48.34	52.09	55.02	32.29	50.56	51.03	71.96	62.66	61.48

The values are mean ± SD (n = 3). NS = not significant (*p* > 0.05). Means with different letters (^a,b,c,…^) are significantly different at *p* < 0.05 within the same row in the parameter. RB and GB denote protein extracts from fully ripe and green KDML 105 rice bran, respectively. Treatments include a non-enzymatic control; single-enzyme extractions using α-amylase (AA) and protease (PT); and sequential extractions (AA-PT and PT-AA).

## Data Availability

The original contributions presented in this study are included in this article. Further inquiries can be directed to the corresponding author.
